# Improvements on speed, stability and field of view in adaptive optics OCT for anterior retinal imaging using a pyramid wavefront sensor

**DOI:** 10.1364/BOE.533451

**Published:** 2024-09-30

**Authors:** Elisabeth Brunner, Laura Kunze, Victoria Laidlaw, Daniel Jodlbauer, Wolfgang Drexler, Ronny Ramlau, Andreas Pollreisz, Michael Pircher

**Affiliations:** 1 Center for Medical Physics and Biomedical Engineering, Medical University of Vienna, Waehringer Guertel 18-20, A-1090 Wien, Austria; 2 Department of Ophthalmology and Optometry, Medical University of Vienna, Waehringer Guertel 18-20, A-1090 Wien, Austria; 3 Johannes Kepler University Linz, Industrial Mathematics Institute, Altenbergerstraße 69, A-4040 Linz, Austria; 4 Johann Radon Institute for Computational and Applied Mathematics, Altenbergerstraße 69, A-4040 Linz, Austria

## Abstract

We present improvements on the adaptive optics (AO) correction method using a pyramid wavefront sensor (P-WFS) and introduce a novel approach for closed-loop focus shifting in retinal imaging. The method’s efficacy is validated through *in vivo* adaptive optics optical coherence tomography (AO-OCT) imaging in both, healthy individuals and patients with diabetic retinopathy. In both study groups, a stable focusing on the anterior retinal layers is achieved. We further report on an improvement in AO loop speed that can be used to expand the imaging area of AO-OCT in the slow scanning direction, largely independent of the eye’s isoplanatic patch. Our representative AO-OCT data reveal microstructural details of the neurosensory retina such as vessel walls and microglia cells that are visualized in single volume data and over an extended field of view. The excellent performance of the P-WFS based AO-OCT imaging in patients suggests good clinical applicability of this technology.

## 1. Introduction

Adaptive optics (AO) assisted retinal imaging allows to visualize the structure and function of the human living retina at cellular and sub-cellular level [1–3]. This plays a crucial role in the investigation of retinal diseases by uncovering potential biomarkers, which give insight in disease mechanisms, progression and the impact of treatment [4,5]. AO has been combined with a manifold of imaging techniques such as fundus camera [6], scanning laser ophthalmoscopy (SLO) [7] and optical coherence tomography (OCT) [8,9]. Major remaining challenges for the clinical translation of AO technology are a more reliable imaging performance in patients and an increase of the small field of view (FoV). The latter is limited by the so called isoplanatic patch [10], an area of ∼1°-2° across where the aberrations introduced by the eye and the imaging system can be assumed constant.

To provide adequate correction of ocular aberrations, the majority of AO supported imaging techniques rely on accurate, fast and robust wavefront sensing [11]. Thereby, the most widespread approach for wavefront sensing in retinal AO incorporates the Shack-Hartman wavefront sensor [12]. Recently, we demonstrated an alternative wavefront sensing method for AO retinal imaging [13]. The concept is based on a non-modulated pyramid wavefront sensor (P-WFS) [14,15] and resulted in high performance AO-OCT imaging of the human retina. The P-WFS based AO showed excellent and highly reliable performance for posterior layer imaging in all subjects and at various imaging locations. However, for reliable imaging of the anterior retinal layers we encountered stability issues, which lowered the applicability of this sensor for this imaging scenario.

In general, for a multilayered structure as the retina, wavefront sensing can be a challenging task, especially in pathological eyes [16,17]. In the healthy (human) eye, the strong backscattering potential of the posterior layers (photoreceptors and retinal pigment epithelium) in comparison to the other retinal layers enables reliable wavefront sensing from this well-defined depth location and facilitates AO correction. However, the situation can be quite different when focusing on different retinal layers (e.g. anterior layers) or in the diseased eye where the normal retinal structure may be heavily distorted.

When the focus of the AO system is set on anterior retinal layers (e.g. retinal nerve fiber layer) light from these layers will significantly contribute to or even dominate the wavefront sensing light. This potentially influences the overall AO correction performance for this focus setting and may lead to un-stable AO correction loops (e.g. when a similar amount of light from the nerve fiber layer and the photoreceptor layer contribute to the wavefront sensing). In the typical approach for focus shifting, a defocus value is added to a target wavefront and the AO-loop converges to this new target [18]. This, however, does not consider the increased light scattering from the anterior layers that is associated with the focus shift.

Another critical aspect for successful AO-imaging is the speed of the AO control loop. The human eye is constantly moving and various processes introduce fast dynamics in the measured wavefront aberrations [19]. To compensate for these dynamics requires AO systems operating at high loop speeds [20,21].

In this work, we improve our concept of AO-OCT retinal imaging on an extended field of view using the non-modulated P-WFS [13]. We increase the AO loop update rate from 5 Hz to 25 Hz and introduce a novel method for closed-loop focus shifting.

We assessed the performance of our improved instrument through imaging of anterior retinal tissue in 10 healthy volunteers and 13 patients suffering from diabetic retinopathy. We show that the faster loop rate reduces the influence of the field aberrations along the slow scanning axis of the AO-OCT system, and effectively increase the size of the high resolution imaging field of view along this axis. Representative AO-OCT image data show an excellent imaging quality obtained with the improvements in both healthy and diseased eyes and highlight the robustness of the proposed AO correction method.

## 2. Method

### 2.1 AO-OCT setup using a pyramid wavefront sensor

**Fig. 1.**
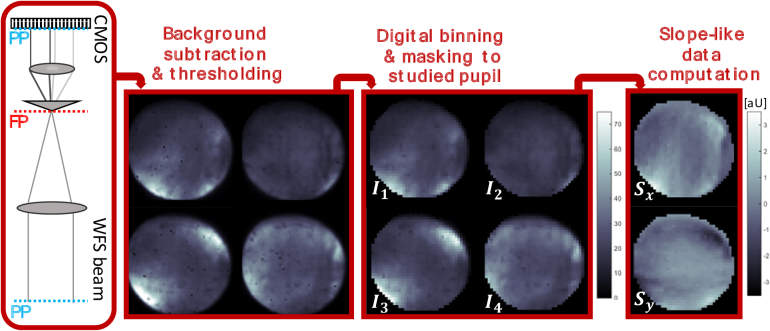
P-WFS schematic and data pipeline (**left to right**): PP marks the pupil plane and FP the focal plane of the system. After sensor read out of the pupil images, background subtraction and intensity thresholding are performed. For digital binning only the illuminated pixels within the studied pupil are included. The P-WFS data consists of two data maps computed from the 4 resulting images according to the standard slope-like data definition. The representative pupil images were recorded *in vivo* in a healthy volunteer.

The instrument used for imaging [13] is a lens-based spectral domain AO-OCT system with a custom-made non-modulated P-WFS and a 69 actuator deformable mirror (DM) from the company ALPAO (Montbonnot, France). The implemented P-WFS consists of a 4-sided glass pyramid in combination with two lenses and a CMOS camera. The pyramid itself is inserted in the wavefront sensing arm of the system at the focal plane of the first lens such that the tip lies in a conjugate retinal plane and splits the light into four parts (cf. [13]). The two lenses of the P-WFS are placed in a 4f configuration such that the input plane is in a plane conjugated to the system’s pupil plane as indicated in the sketch of Fig. 1. The images of the sensor are formed in the back-focal plane of the second lens (pupil plane) and are recorded by the detector. Differing intensity distributions within the four images are observed when wavefront aberrations are present (cf. Figure 1). After the camera read out, the four pupil images are processed with the customized P-WFS data pipeline [13] visualized in Fig. 1. The two slope-like data maps are computed according to the standard model

(1) $$S_{x}=\frac{1}{I_{0}}[\left(I_{1}+I_{4})-(I_{2}+I_{3}\right)],$$

(2) $$S_{y}=\frac{1}{I_{0}}[\left(I_{1}+I_{2})-(I_{3}+I_{4}\right)],$$

where $I_{i}$ for i=1,…,4 are the pupil images as depicted in Fig. 1 and $I_{0}$ is the mean intensity in the four pupil images.

The calibration of closed-loop AO control with a P-WFS is performed in a model eye which consists of a lens and a scattering surface while the scanners are moving with a small scanning angle. For each DM actuator, the sensor response for a pushing action with 10% of the maximum stroke is recorded. A reference frame is acquired for a flat DM and subtracted from the poking P-WFS data. The resulting slope-like data maps are used to form the DM to P-WFS interaction matrix. The command matrix is obtained via truncated singular value decomposition (SVD) of the interaction matrix. The closed-loop actuator command update uses a standard proportional-integrator control [22]:

(3) $$c_{update}(i)=CM(S(i)-S_{target}),$$

(4) $$c_{DM}(i+1)=c_{DM}(i)+\delta \;c_{update}(i),$$

where $S(i)=[S_{x}(i);S_{y}(i)]$ is the residual WFS slope data measured in loop iteration *i* and CM denotes the command matrix. A constant loop gain δ was applied. The residual WFS slopes are driven towards a pair of target slopes $S_{target}=[S_{target,x};S_{target,y}]$ , which are set to zero. This standard calibration procedure was described previously [13] and reliably provides stable and precise closed-loop AO correction when the focus is set to the photoreceptor layer for standard field of views (1°-2°) and extended field of views of up to 4°.

### 2.2 In vivo calibration procedure for P-WFS based closed-loop focus shifting

In order to shift the focus in closed-loop condition, the zero slope target in Eq. (3) is replaced by a slope target that represents a defocus wavefront. The target slopes are commonly obtained by computing theoretical defocus slopes. In AO systems using the SH-WFS, the required slope maps are directly calculated from the desired defocus wavefront.

**Fig. 2.**
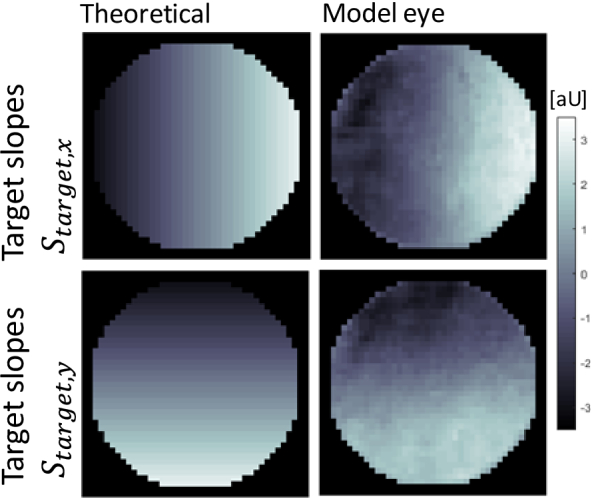
Comparison of target P-WFS slope maps for a defocus wavefront that were theoretically computed and measured in the model eye. A root mean square (RMS) value of -0.8 µm was used.

Because our P-WFS approach is not based on wavefront reconstruction, a different approach is needed. Within the slope-like P-WFS data definition, a defocus wavefront is represented in a tip and tilt of the x- and y-direction slope data, respectively, as depicted in Fig. 2. However, residual alignment errors in the P-WFS may cause a slight rotation of the four pupil images. In addition, an uneven distribution of the light power amongst the four pupil images may occur. These effects are not sufficiently accounted for by the theoretical focus shifting approach and a closed-loop correction will be diverging. An alternative approach is to record target images in the model eye for different amounts of defocus values (introduced by a deformable mirror, see Fig. 2). However, it turns out that this approach is not stable when applied to *in vivo* imaging. A possible explanation lies in the structural discrepancies between the model eye and the *in vivo* eye. While the artificial retina consists of a single layer, the human retina is a multi-layered structure that varies between individuals and with the retinal imaging location.

To account for these issues, we implemented an *in vivo* calibration procedure for stable closed-loop focus shifting. This procedure is performed pre-imaging for each subject, at each imaging location and for each defocus level. The automated procedure starts with closing the AO loop until convergence is achieved without focus shifting. The loop is shortly opened, the current DM shape stored and the desired defocus wavefront is added to the corrective DM shape already in place. P-WFS pupil images are recorded for this focus setting and slope-like data maps are computed using the P-WFS data pipeline. The stored DM shape is loaded and the loop is now closed on the new P-WFS target slopes corresponding to the chosen defocus value.

One key factor for this procedure is the correct application of the desired defocus value on the DM. Thus, we calibrated the feed-forward control matrix used for the application of the defocus wavefront. The details of this procedure are described in Supplement 1 (Section S1)**.** Through this calibration we found a good linear relationship between set defocus values on the DM and measured wavefront in the pupil plane.

**Fig. 3.**
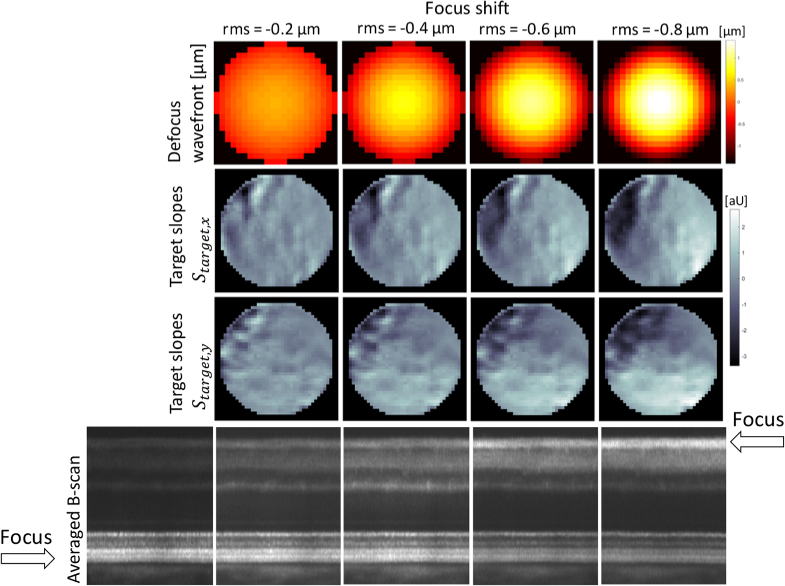
Representative image data of a closed-loop focus shifting calibration. **First row**: Defocus wavefronts of -0.2, -0.4, -0.6 and 0.8 µm RMS value, respectively, introduced by the deformable mirror during calibration of the closed-loop focus shifting. **Second/third row**: P-WFS target slope maps in x/y direction recorded *in vivo* for the respective defocus wavefronts. **Fourth row**: Averaged B-scans extracted from AO-OCT volumes recorded with the focus of the AO correction set according to the respective defocus target. The representative calibration and imaging data were recorded 14° temporal / 6° superior in a healthy volunteer (f, 27 years) with the scanners forming a FoV of ∼1°×1°. The AO-OCT data on the far most left was recorded without focus shifting.

With defocus targets obtained by this *in vivo* calibration, stable and precise focus shifting is achieved. In Fig. 3, representative image data for a focus shifting calibration run performed in a healthy volunteer are provided. In the averaged B-scans, anterior retinal layers appear with increased intensity according to the respective defocus level.

One aspect of the above procedure is the scanning frequency along the slow axis. During convergence of the AO correction, the sampling in this direction is reduced such that the WFS measurements are averaged across a large part of the FoV [13]. The same scanning pattern is applied during focus shifting calibration. To minimize remaining effects of the slow scanner motion on the P-WFS data, it has proven beneficial to use an average of a small number of P-WFS frames (usually three) as target slopes.

### 2.3 Improved AO loop update rate

To decrease the impact of accommodation and eye movements, the P-WFS exposure time was reduced from 40 to 20 ms, and the control implemented in C/C++. This resulted in a five-fold increase in AO loop rate from 5 to 25 Hz. With the achieved speed up, the execution time of the focus shifting calibration for one defocus level could be reduced from ∼825 ms to ∼175 ms, which is a key factor for a broad applicability of the method.

The impact of the improved speed on the convergence time and the correction bandwidth of the AO loop was analyzed for a 4°×4° FoV both in the model eye and *in vivo.* For this purpose, time series were recorded containing the overall RMS value of both P-WFS slope maps for each loop iteration. Since the loop time shows fluctuations (because of an operating environment in Windows 10), additionally the time point of each iteration was saved. Based on this loop time information, the RMS values were interpolated to create time series with equidistant time steps of 60 ms. It has to be noted that no wavefront reconstruction was performed and that for larger aberrations the P-WFS slope maps do not scale linearly with the wavefront aberrations [14,15,23]. Therefore, the RMS value of the P-WFS slope maps does not give an absolute but only a relative measure of the (residual) wavefront aberration strength. Nevertheless, the recorded time series provide insight into the temporal behavior of the (residual) wavefront aberrations present.

For the experiments in the model eye, the same constant loop gain of 0.5 and exposure time of 20 ms which is also applied during *in vivo* imaging were used. Since the artificial retina of the model eye shows stronger scattering than the human retina a neutral density filter (NF D1.3) was placed in the collimated beam directly after the entrance collimator of the light source to obtain similar power incident at the WFS as in the *in vivo* imaging scenario.

#### 2.3.1 Analysis of AO loop convergence time

For *in vivo* imaging, the number of B-scans in slow scanning direction is reduced during AO convergence to average the wavefront measured in one WFS exposure over a large part of the FoV. Consequently, we use the sparse scanning pattern for the assessment of the convergence time of the AO correction and the closed-loop focus shifting.

We defined the convergence time of the P-WFS based AO correction as the time span between the moment the loop was closed and the moment a RMS value lies below the averaged RMS convergence value. The experiment was conducted in the model eye for the original loop update rate of 5 Hz and the improved update rate of 25 Hz. Through axial displacements of the artificial retina, myopic and hyperopic eyes were modelled. Thereby, defocus aberrations of ±0.5, ± 1 and ±2 Diopter were introduced. The measurement was repeated 5 times for each defocus level and the results were averaged. For the faster update rate of 25 Hz, the experiment was further performed in four healthy volunteers. For the *in vivo* experiment, four measurements were recorded and averaged in each subject.

The convergence time of the closed-loop focus shifting was obtained in a similar fashion. Here, we switched between a zero target and the calibrated defocus target (-0.8 µm RMS value). The focus shifting convergence time was determined as the time span from the target switch to the AO loop convergence as described above. The experiment was performed in the model eye for the same Diopter settings and in the same four healthy volunteers, with three and two repetitions, for the model eye and the *in vivo* measurements, respectively.

#### 2.3.2 Analysis of AO correction bandwidth

For the analysis of the AO correction bandwidth, the 4°×4° FoV is scanned at full resolution of 750 lines as is the case when recording *in vivo* image data. The goal is to measure the temporal variations of the wavefront under open- and closed-loop conditions, similar to previous work [20]. However, in our case we additionally encounter aberrations introduced by the large FoV. In order to avoid a bias in the RMS time series that is caused by the absolute RMS value, the open-loop data was recorded after static aberration correction. This is obtained by simply opening the AO loop after convergence of the closed-loop correction. Otherwise the noise characteristics between open- and closed-loop are not comparable because the lower RMS values of the closed-loop operation always show lower noise.

Each time series covers a duration of 18 seconds corresponding to ∼90 and ∼450 loop iterations at update rate 5 Hz and 25 Hz, respectively. The experiment was performed in the model eye in the presence of a defocus wavefront of +0.5 Diopter for both update rates. For the faster update rate, the experiment was repeated in three healthy volunteers. For each setting, four time series were recorded for the static correction and closed-loop correction scenario. After subtraction of the mean RMS of a series, power spectra were computed for the open- and closed-loop operations and averaged [20]. By computing the quotient of the respective averaged closed-loop and static correction power spectra, power rejection curves were obtained. The cut-off frequency of the AO correction can now be retrieved as the frequency value at which the power rejection curve becomes larger than one [24].

### 2.4 Subject selection and imaging protocol

Ten healthy subjects aged between 27 and 67 years and thirteen patients aged between 37 and 80 years suffering from non-proliferative as well as proliferative diabetic retinopathy (DR) have been imaged with the improved AO. The subjects were recruited with the selection criteria of acceptable media opacities and fixation capabilities. All measurements adhered to the tenets of the Declaration of Helsinki and were performed after approval of the study by the local ethics committee. Written consent was obtained from each subject prior to the measurement after explaining the nature and form of the procedures.

A headrest and a manually adjustable XYZ translation stage were used to stabilize and align the heads of the subjects in respect to the instrument and the subjects were asked to fixate with the eye being imaged on an external fixation target. For pupil dilation and suppression of accommodation, Tropicamid was administered ∼20 minutes prior to imaging. Due to the large age range and the fact that several patients had artificial lenses, the pupil diameters were nevertheless varying between 5 mm and 8 mm. For the four youngest healthy volunteers V1 (f, 28 y, -1.00 + 0.50 Diopter), V2 (f, 27 y, -1.25 + 0.75 Diopter), V3 (m, 29 y, -0.50 + 0.75 Diopter) and V4 (m, 24 y, -0.75 + 0.50 Diopter), no drugs were used since their pupil diameter was larger than 6 mm under the low light conditions in the lab.

The system provides a 250 kHz A-scan (depth profile) rate and supports scanning angles of up to 4°×4°. The theoretical axial resolution is 4.5 µm in tissue at 840 nm imaging wavelength with 50 nm bandwidth. With a design pupil of 7 mm in diameter, the system has a theoretical transverse resolution of ∼2 µm. The AO control algorithm automatically adapts to the observed pupil size of the subject. The presented AO-OCT volume data were recorded at an extended FoV using scanning angles of 4°×4°, which corresponds to an imaging area of ∼1.4 × 1.4 mm on the retina. A sampling density of 750 × 750 pixels (750 A-scans per B-scan, 750 B-scans) in x and y direction has shown to provide good visualization of the targeted structures in the anterior retinal layers while minimizing motion artifacts. The sampling in z- (depth) direction is 400 pixels (corresponding to ∼2.36 mm imaging depth) according to the used spectrometer configuration (the light spectrum is dispersed over 800 pixels). The en-face and cross-sectional images provided in the result and discussion sections were extracted from single-acquisition AO-OCT volumes with a recording time of ∼2.5 seconds per volume at a B-scan rate of a ∼300 frames per second. No volume averaging was applied.

Five to nine imaging locations and three to five focus positions per imaging locations were included in each imaging session. The duration of the imaging sessions was typically between 10 and 30 minutes. After convergence of the AO correction, the target calibration for the chosen focus shift was performed prior to every single data acquisition, which consists of two AO-OCT volumes recorded in a row. For every defocus target calibration, a pair of raw P-WFS images was stored, with one image recorded before the defocus wavefront is applied by the DM and a second image after. The latter is then used for the computation of the target slopes which are directly employed in the subsequent focus shifting.

### 2.5 Data processing

The processing applied to the recorded spectral data is similar to standard OCT processing [25,26] and images are shown on a linear intensity grey scale. For all data volumes, rotation of the B-scan images and curvature of the retina within the B-scans were compensated by adding corrective linear phase terms to the spectral data as described in detail elsewhere [27]. Lateral motion between the B-scans and axial displacement between B-scans were corrected in post processing. The axial displacement between B-scans was determined by averaging all A-scans laterally in each B-scan and computing the cross-correlation of the averaged A-scan profiles of all B-scans with the profile of the central B-scan. The robustness of the axial correction against the presence of large vessels could be increased by cropping the averaged A-scan profiles to either the anterior or the posterior layers. Depending on the retinal section chosen for the cropped area, the B-scans will be aligned either along the anterior or the posterior layers.

When visualizing a specific layer, a surface fit to that layer was performed and the A-scans were then aligned to that layer.

## 3. Results

The performance of the improved P-WFS based AO correction was assessed in the model eye and *in vivo.* All *in vivo* data presented was recorded at imaging locations along the superior vessel arc to test the robustness of our focusing method in the presence of a thick nerve fiber layer and large vessels.

### 3.1 AO convergence analysis

The convergence times for the 5 and 25 Hz AO-loop operation are presented in Table 1. A dependence of the convergence time on the aberration strength can be observed. This can be explained with the non-linearity of the sensor response and the fact that a constant loop gain of 0.5 was applied. The *in vivo* results are in accordance to the model eye measurements. For the fast update rate, we measured averaged convergence times between ∼0.5 and ∼1.5 seconds. Eye motion, blinks and accommodation, which was not suppressed in this experiment, as well as the smaller number of measurements included explains the larger standard deviation observed for the *in vivo* results.

**Table 1.** Convergence times of closed-loop AO correction and closed-loop focus shifting. The times are given in ms and are measured in the model eye for varying amounts of defocus aberrations present for 5 Hz and 25 Hz AO loop update rate (top) and *in vivo* in four healthy subjects for 25 Hz AO loop update rate (bottom). As data points we provide (mean|std) pairs computed for repeated measurements.

Model eye defocus			+0.5 D	-0.5 D	+1 D	-1 D	+2 D	-2 D
AO correction	5Hz	mean	1197	1442	1966	2184	6219	6167
		std	230	307	340	124	135	663
	25Hz	mean	308	336	299	4494	1127	1135
		std	81	33	37	64	80	41
Focus shifting	5Hz	mean	6167	5072	6034	3138	6356	3713
		std	239	522	226	616	1032	1030
	25Hz	mean	548	459	574	438	599	247
		std	55	31	77	64	6	67

Volunteer			Subject V1	Subject V2	Subject V3	Subject V4
AO correction	25Hz	mean	465	1478	1042	916
		std	39	297	152	78
Focus shifting	25Hz	mean	279	562	424	512
		std	38	30	60	69

For the convergence time of the closed-loop focus shifting, no dependence on the present aberration strength prior to AO correction was observed. These aberrations are already pre-corrected in the first step (closed-loop without defocus) and results to a faster convergence within ∼0.2–0.6 seconds in the model eye and in the healthy volunteers.

### 3.2 Analysis of the AO bandwidth and of the aberrations across the field of view

**Fig. 4.**
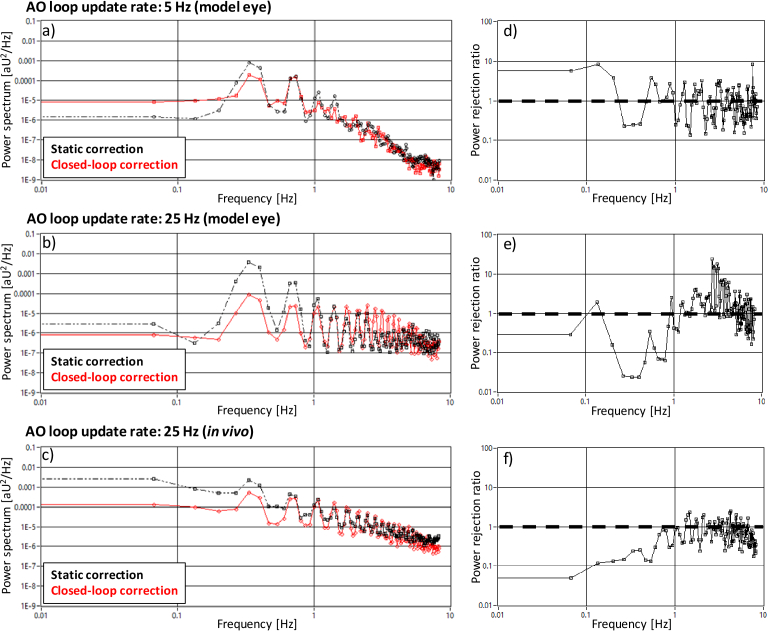
Analysis of the AO-bandwidth in the model eye and in-vivo. **a-c)** Power spectra computed for a time series (18 seconds) of RMS values of P-WFS slope maps recorded for static aberration correction (black) and closed-loop correction (red). **d-f)** Power rejection curves computed as quotient of the respective closed-loop correction and static correction power spectra. The model eye data are an average of four recordings. The *in vivo* data was obtained from 3 healthy volunteers and 4 time series per volunteer.

The recorded time series of RMS values of the PWS-slopes are displayed in Supplement 1, Fig. S2. The retrieved averaged power spectra for both open- and closed- loop conditions are shown in the left column of Fig. 4. Scanning and aberrations across the field of view introduce temporal fluctuations at the corresponding scanning frequency (∼0.4 Hz of the slow scanner). In addition, higher harmonics are visible as well that are probably introduced by the fast resetting of the scanner to its original position after performing a scan. To further emphasize the influence of the scanning onto the power spectra, additional analysis and results of varying scanning protocols are shown in Supplement 1 (Section S2).

The original loop update rate of 5 Hz is not fast enough to significantly suppress the temporal dynamics introduced by the slow scanner, as can be seen in Fig. 4(a)). In contrary, the AO loop introduces additional variations within the FoV in the residual wavefront, e.g. due to overshooting of the correction. This causes the larger amplitudes at low frequencies in the power spectrum of the closed-loop case in comparison to the open-loop. With the improved loop update rate of 25 Hz however, the influence of the field aberrations in the slow axis is clearly reduced (cf. Figure 4(b))). This finding is also reflected in corresponding power rejection curves in Figs. 4(d)) and (e). With the improved update rate, a cut-off frequency of ∼1 Hz is obtained for the AO correction indicating the true bandwidth of our AO loop. The results could be confirmed *in vivo* with AO data recorded in three subjects as shown in the power spectra and the resulting power rejection curve in Fig. 4(c)) and f), respectively.

**Fig. 5.**
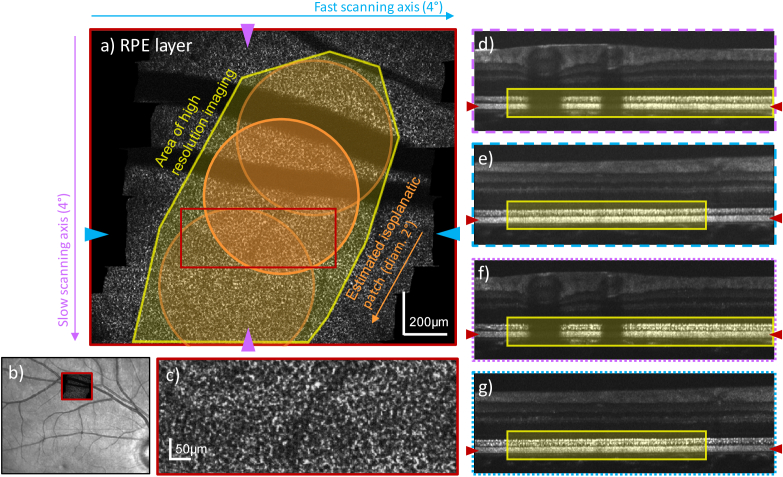
Representative AO-OCT data recorded with a field of view of 4°×4° (corresponding to 1.46 × 1.46 mm on the retina) in the superior vessel arc of a healthy volunteer (female, 27 years, right eye, 6.38 mm pupil diameter). The focus was set to the photoreceptor layer. The en-face image in **a**) was retrieved from a single data volume by depth integration over the retinal pigment epithelium (RPE). The location of the zoom-in shown in **c**) is indicated with a red box in **a**). Cross-sectional views were extracted along the slow (purple) and fast (blue) scanning axis. The average and the standard deviation of 50 adjacent B-scans are shown in **d**),**e**) and **f**),**g**), respectively. The locations at which the en-face and cross-sectional images were extracted are marked by color coded arrows in **d**)-**g**) and **a**), respectively. The AO-OCT imaging location is marked on a Spectralis infrared fundus image in **b**). The estimated size of the isoplanatic patch is indicated by orange circles in **a**) and the approximated area of high resolution imaging is marked in yellow in **a**), **d**)-**g**).

To demonstrate the benefit of the higher AO loop rate for reducing aberrations across the slow-scan direction we recorded *in vivo* data with focus on the posterior retinal layers. Figure 5 shows representative en-face views of the RPE and averaged B-scans in the fast and slow scan directions, respectively. In this example, we define the area of high resolution imaging as the area in the RPE en-face image where the mosaic is clearly visible. There are various criteria to define the isoplanatic patch in human eyes, but the literature agrees that for pupil sizes of 6 mm or larger it is typically not larger than 2° in diameter [10]. Therefore, without compensating field aberrations, one would expect a visibility of the RPE mosaic in Fig. 5(a)) only within the 2° orange circle at the center of the field of view. However, since the AO loop is fast enough to correct for the changes of the wavefront along the slow scanning axis, the isoplanatic patch moves along with the imaging beam. Thus, the area of high resolution imaging is extended along this scanning axis (area that is marked in yellow in Fig. 5(a)). This effect cannot be observed along the fast scanning axis, since the wavefront measurement is averaged along this scanning direction.

The same observation can be made in the cross-sectional views in Figs. 5(d))-g) which were computed along the slow and fast scanning axis, respectively. In the averaged B-scan of Fig. 5(d)), the intensity variation within the photoreceptor band along the slow scanning axis is much less pronounced than along the fast scanning axis in Fig. 5(e)). The same observation holds for loss of contrast in the photoreceptor band along the slow and fast scanning direction as measured by the standard deviation between the B-scans (cf. Figure 5(f)) and g)). At the beginning of the volume, the visibility of the RPE mosaic in the en-face image Fig. 5(a)) deteriorates. This can also be seen by the lower intensity and contrast of the photoreceptor band in the B-scans (Fig. 5(d)) and g)). The jump of the slow axis scanner, which introduces a residual error in the AO correction is the most likely cause for this observation.

Visualization 1 provides an en-face fly-through of the posterior retinal layers and additional en-face images of the photoreceptor bands extracted from the same volume are shown in Fig. S6 of Supplement 1.

### 3.3 Anterior layer AO-OCT imaging in healthy and pathological eyes

Stable focus shifting while maintaining good AO correction was achieved in all subjects for the duration of ∼5 seconds. This is the approximate imaging time required to record two AO-OCT volumes at an extended FoV. An analysis of the stability of the focus shifting for longer periods of time (∼16 seconds) is provided in Supplement 1 (Fig. S7 of Section S4).

In this section, representative AO-OCT data acquired with two different focus shifts (at nerve fiber layer and inner limiting membrane) are shown. Images of the retina of the same healthy volunteer V2, which are of excellent AO correction quality, are serving as a benchmark for imaging quality achievable in patients with diabetic retinopathy. All en-face and B-scan images are extracted from single acquisition volumes by depth and cross-sectional integration of image frames, respectively.

**Fig. 6.**
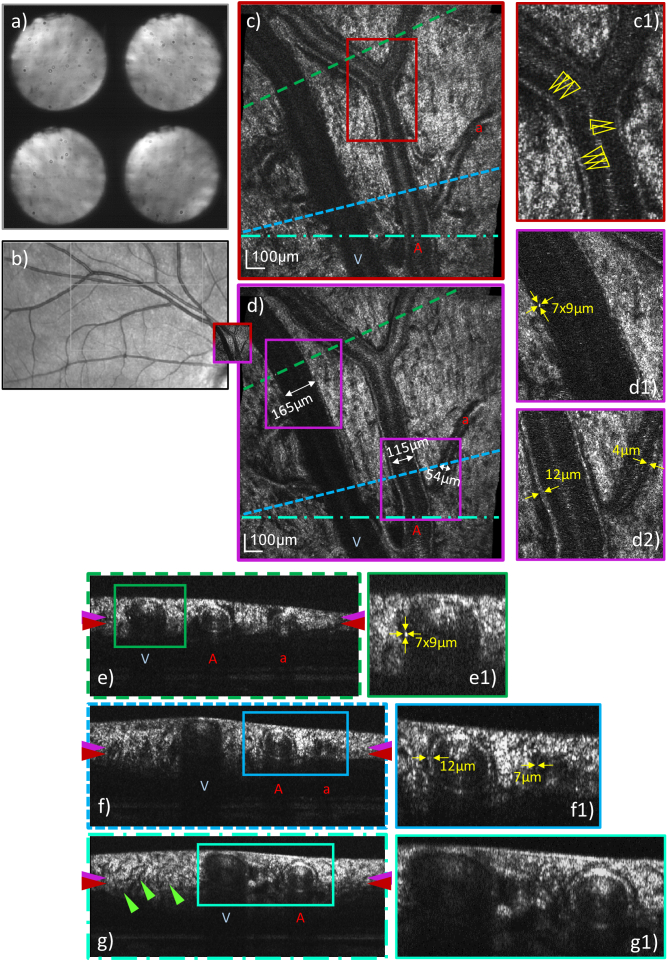
Representative AO-OCT data visualizing retinal vasculature recorded close to the optic disc in a healthy volunteer (27 years, female, right eye, 6.3 mm pupil diameter) with a FoV of 4°×4° and the focus set to the nerve fiber layer. Pupil plane images recorded by the P-WFS are shown in **a**). The AO-OCT imaging location is marked on a Spectralis infrared fundus image in **b**). The en-face images in **c**)**-d**) and B-scans in **e**)**-g**) are extracted from a single data volume by depth and cross-sectional integration of image frames, respectively. B-scan locations are marked by color-coded dashed lines in **c**)**-d**), depth locations of en-face images by color-coded arrows on the sides of **e**)**-g**). Vessel types are indicated with letters *V* for vein, *A* for artery and *a* for arteriole. The wall-to-wall diameters of the three considered vessels are given in **d**). In the zoom-ins **d2**) and **f1**), vessel wall thickness of the artery and arteriole are provided. In **d1**) and **e1**) the same hyper-reflective microstructural detail of the vein wall is measured in size. Representative locations of where the arterial wall appears multi-layered are indicated in **c1**) by yellow arrows. The green arrows in **g**) point at the pocketed arrangement of the nerve fiber bundles just before entering the optic disc.

**Fig. 7.**
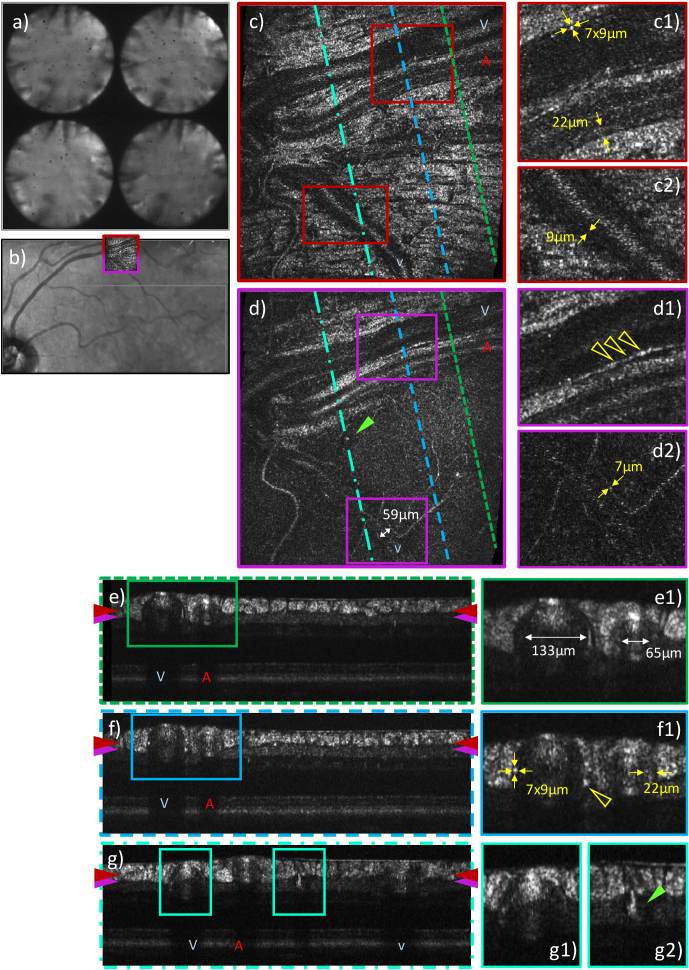
Representative AO-OCT data visualizing retinal vasculature recorded in the superior vessel arc of a patient with moderate DR (57 years, male, left eye, 6.94 mm pupil diameter) with a FoV of 4°×4° and the focus set to the nerve fiber layer. The P-WFS pupil plane images in **a**) show intensity variations introduced by cataract. **b**) The AO-OCT imaging location is marked in a Spectralis infrared fundus image. The en-face images in **c**)**-d**) and B-scans in **e**)**-g**) are extracted from a single data volume by depth and cross-sectional integration of image frames, respectively. B-scan locations are marked by color-coded dashed lines in **c**)**-d**), depth locations of en-face images by color-coded arrows on the sides of **e)-g**). Vessel types are indicated with letters *V* for vein, *A* for artery and *v* for venule. The wall-to-wall diameters of the three considered vessels are given in **d2**) and **e1**). The vessel wall thickness of the artery and venule are provided in the zoom-ins **c1**) and **f1**), and **c2**) and **d2**), respectively. In **c1**) and **f1**) the same hyper-reflective microstructural detail of the vein wall is measured. The yellow arrows in **d1**) and **f1**) point at a hyper-reflective band within or adjacent to the vein wall. The green arrows in **d**) and **g2**) indicate the location of a micro-aneurysm.

With the focus set to the nerve fiber layer, the walls of retinal vasculature [28,29] are visualized for a healthy subject (cf. Figure 6), and for a 57-year-old patient with moderate diabetic retinopathy in (cf. Figure 7). The AO-OCT imaging locations are marked in a Spectralis infrared fundus image in Figs. 6(b)) and 7(b), respectively. Full resolution pupil plane images recorded by the P-WFS after loop convergence and prior to the *in vivo* focus target calibration allow for an assessment of the pupil plane. The healthy subject has clear optical media and the pupil shows only minor obstructions through eye-lashes on the top of the lid, as seen in Fig. 6(a)). The pupil images of the patient on the other hand are of reduced intensity and show finger- like shadowing from the patient’s cataract reaching far into the pupil. Nevertheless, comparable AO correction quality was achieved in both subjects and similar details of the vessel walls can be observed in en-face images in Figs. 6(c)-(d) and Figs. 7(c)-(d), as well as in the cross-sectional views in Figs. 6(e)-(g) and Figs. 7(e)-(g).

In Visualization 2 and Visualization 3, an en-face fly-through of the anterior retinal layers and a movie of all B-scans are shown for the representative data recorded in the healthy volunteer. A movie showing an en-face fly-through of the anterior layers of the patient is provided in Visualization 4.

**Fig. 8.**
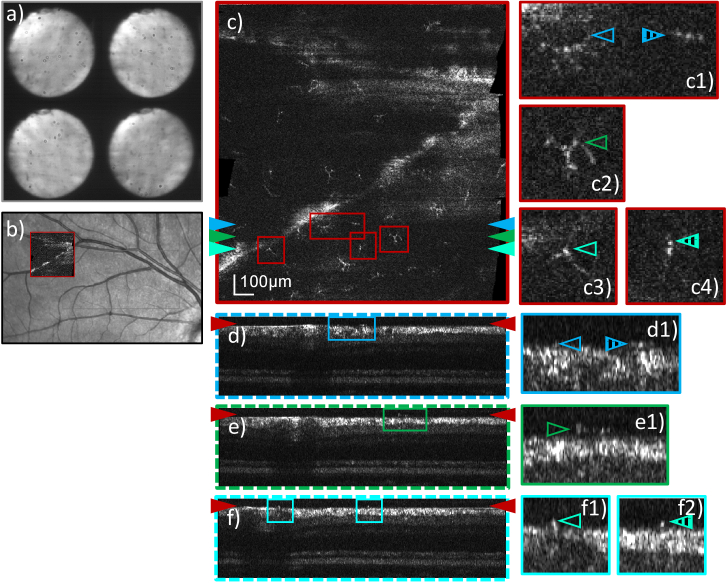
Representative AO-OCT data visualizing microglia recorded in the superior vessel arc of a healthy volunteer (27 years, female, right eye, 6.43 mm pupil diameter) with a FoV of 4°×4° and the focus set to the inner limiting membrane. Pupil plane images recorded by the P-WFS are shown in **a**). The AO-OCT imaging location is marked in a Spectralis infrared fundus image **b**). The en-face image in **c**) and B-scans in **d**)-**f**) are extracted from a single data volume by depth and cross-sectional integration of image frames, respectively. B-scan locations and depth location of the en-face image are marked by color-coded arrows on the sides of **c**) and **d**)**-f**), respectively. Zoom-ins on selected microglia cells are provided in both en-face and cross-sectional view, in which corresponding details are marked by (color and appearance) matching arrows.

**Fig. 9.**
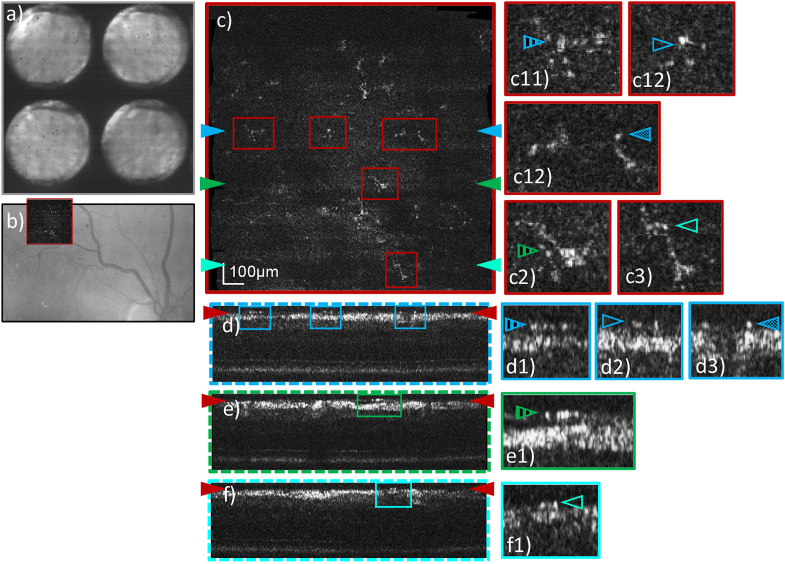
Representative AO-OCT data visualizing microglia recorded in the superior vessel arc of a patient with severe DR (67 years, male, right eye, 6.54 mm pupil diameter) with a FoV of 4°×4° and the focus set to the nerve fiber layer. The P-WFS pupil plane images in **a**) show intensity variations introduced by an artificial lens. The AO-OCT imaging location is marked in a Spectralis infrared fundus image **b**). The en-face image in **c**) and B-scans in **d**)**-f**) are extracted from a single data volume by depth and cross-sectional integration of image frames, respectively. B-scan locations and depth location of the en-face image are marked by color-coded arrows on the sides of **c**) and **d**)**-f**), respectively. Zoom-ins on selected microglia cells are provided in both en-face and cross-sectional view, in which corresponding details are marked by matching arrows.

In a next step the focus was placed to the inner limiting membrane. Imaging was performed in a healthy subject and in a 67-year-old patient suffering from severe diabetic retinopathy (cf. Figures 8 and 9). In the case of the patient the borders of an artificial lens are clearly visible in the pupil images (cf. Figure 9(a)). Nevertheless, in both cases not only the cell bodies but also the processes (ramifications) of microglia cells [30–32] can be visualized (cf. Figures 8(c) and 9(c)). This allows for an analysis of the appearance and the spatial distribution of these microglial cells. The positioning of cell bodies and processes with respect to the inner limiting membrane can be understood through the cross-sectional images provided in Figs. 8(d)-(f)) and Figs. 9(d))-f).

## 4. Discussion

AO correction (without focus shifting) targets to minimize the measured wavefront error of the light returning from the retina, i.e. a zero slope target is used for the AO control loop. This usually coincides with a focus of the imaging beam set within the retina on the RPE/photoreceptor band because these layers are highly reflective/backscattering and thus light primarily from these layers will contribute to the wavefront sensor image.

Changing of this focus setting requires a change in the target wavefront that is typically computed theoretically or recorded in a model eye (according to private conversation with various groups using the SH-WFS). However, our observation using a pyramid wavefront sensor indicated that this approach was suboptimal. The AO-loop frequently became unstable or even failed to converge. One reason is that the computed defocus targets seem not representative of the real WFS measurement that is recorded when a focus shift is present. For example, due to the focus shift, the measurement will show increased light contributions from other retinal layers such as the RNFL that are located at a different imaging depth and thus will influence the measured wavefront. Another aspect are non-linearities that are quite pronounced in case of a pyramid wavefront sensor [23]. These possibly influence the AO-correction in the presence of stronger (defocus) aberrations as is the case with the intentional focus shift.

To overcome these issues, we introduced a new method for stable closed-loop AO operation at a shifted focus position. The method requires an initial closed-loop correction of ocular aberrations with focus on the RPE that is followed by an *in vivo* calibration step for the specific focus setting. Thereby, the desired defocus wavefront is introduced by the DM and the resulting P-WFS slope maps at this focus position are recorded. These slope maps are then used as target for closed-loop operation at the shifted focus position. As outlined for example in Figs. 6 to 9, this approach yields excellent image quality of the anterior retinal layers in healthy subjects and patients. Furthermore, the method showed good stability over a total time span of ∼16 seconds (cf. Fig. S7 in Supplement 1). It should be noted that this new focus shifting method can be easily translated to the AO control of other wavefront sensors, such as the SH-WFS, and to other AO retinal imaging modalities, such as AO-SLO, as well, potentially improving AO performance.

Prerequisite for the proposed calibration step is excellent control of defocus introduced by the DM and a fast AO control loop. As shown in Fig. S1 of Supplement 1, the used DM showed good linearity between set and measured defocus values. This indicates a good reliability of the set defocus values during our calibration step. Our improved loop speed of 25 Hz results in a duration of the calibration of ∼175 ms per defocus level, which is fast enough to minimize influences of eye motion or accommodation.

A further improvement of the wavefront sensing and AO correction speed is in principle possible [20,21] and would further reduce these influences. The upper limit in terms of AO correction speed that can be achieved depends on the exposure time of the wavefront sensor that is needed to obtain sufficient signal to noise ratio and the computation time of the control loop. Both can be improved, for example, by implementing the pixel binning that is currently performed in post-processing on the camera itself.

Our analysis summarized in Table 1 showed clear benefits of a faster loop speed in terms of convergence time and thus AO correction stability. Depending on the strength of the present wavefront aberrations, the AO correction reaches convergence after 0.3–1.5 seconds. This greatly facilitates subject imaging because image recording can be started immediately after alignment and after blinks. In our experience, a faster AO loop speed is beneficial for translating AO retinal imaging with the P-WFS to clinical applications. Patient imaging is often performed in elderly subjects, who present larger ocular aberrations and stronger eye movements. In addition dry eyes, leading to more frequent blinking and changes in the tear film, are very common [33,34]. With the faster convergence and improved stability of the AO correction, specifically for anterior layers, we could include more imaging locations and focus settings in DR patients in a shorter time frame. This allows for the investigation of microstructural details of the retina over a larger imaging area even in patients.

Another aspect that has not yet been employed in our setup is the calibration of the influence matrix at predefined defocus levels. It has been shown that the influence matrix has some dependency on the overall deformation of the mirror (such as defocus) [35]. To improve the AO performance, the use of an influence matrix corresponding to a specific focus settings is ideal. Although, an *in vivo* calibration seems not feasible (because of motion artifacts and the long duration of the procedure) such calibration can be in principle performed in the model eye. This allows for the optimum influence matrix to be used for a specific focus setting, potentially further improving the AO-performance.

An important aspect of our system is the observed reduction of the influence of field aberrations along the slow scanning axis for the 4°×4° FoV (as shown in Fig. 5). This is enabled by our specific wavefront sensing method that uses the same light for imaging and wavefront sensing and the higher AO correction bandwidth. The latter enables to decrease the distance between the areas where the wavefront is measured and corrected. Good correction is enabled when both areas still lie within the isoplanatic patch. This observation elevates typical AO imaging field of view restrictions that are otherwise determined by the isoplanatic patch of the individual eye. Some approaches have been introduced to increase the field of view such as multi-conjugate adaptive optics [36] or the exploitation of spatially incoherent illumination in full field OCT [37], but these either add to system complexity or are limited to specific imaging configurations.

The exploitation of a fast AO update along the slow scanning direction seems easier to be implemented. Furthermore, this principle can be translated to various AO-systems including SLO to cover an even larger field of view in a single acquisition. However, for SLO systems the correction speed needs to be much higher to account for the faster y-scanning speed and the wavefront sensing beam needs to be scanning in accordance to the imaging beam.

With our system we successfully imaged 10 healthy volunteers and 13 patients with diabetic retinopathy (non-proliferative as well as proliferative). In all healthy subjects we observed stable focus shifting and excellent AO correction performance. This was determined by detailed visual inspection of all the recorded volumes. From the 13 patients, 13 eyes of 10 patients yielded similar imaging quality as achieved in the healthy eyes. 7 eyes (5 patients) showed degraded AO performance, while 6 eyes were excluded because of surgery or too opaque media. Imaging locations were chosen along the superior vessel arc, where thick nerve fiber bundles and large retinal vessels are prevalent. These locations typically represent challenging locations for AO correction and our results support the feasibility and applicability of the technique.

**Fig. 10.**
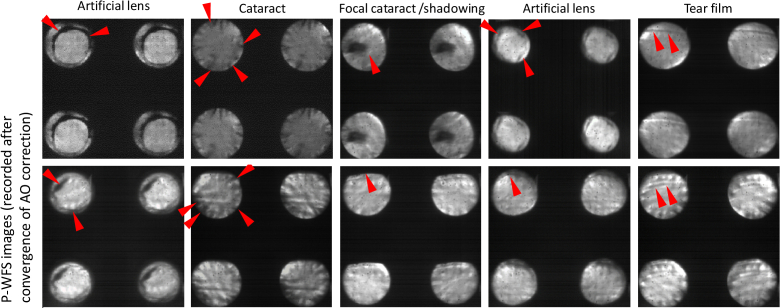
A representative collection of full resolution pupil plane images recorded for 10 eyes of patients suffering from diabetic retinopathy by the P-WFS after convergence of the AO correction loop. Intensity variations which are not due to wavefront aberrations are marked with red arrows.

Imaging of this retinal location requires a fixation target above the horizontal plane of the system and the imaging beam enters the pupil close to the upper eye lid during imaging. This frequently leads to obstructions of the beam at the pupil edges by eyelashes and sometimes by the eye lid itself. For patients, this often comes in combination with smaller and irregular shaped pupils. The strong variations in pupil size and shape amongst the patients that have been included in the study can be seen in the representative full resolution pupil plane images recorded with the P-WFS (cf. Figure 10).

The presence of these intensity variations that are not caused by wavefront aberrations but by cataract, artificial lenses and changes in the tear film (cf. Figure 10) potentially influences the pyramid wavefront sensor measurements (as this sensor retrieves the waverfront slopes from the recorded intensity pattern). However, the AO correction maintained good performance when using the new focus shifting method because these intensity fluctuations are directly incorporated into the recorded defocus targets. One requirement though are fairly stable pupil positions, otherwise the convergence of the AO is less stable.

The access to the full resolution pupil images certainly represents an advantage of the pyramid wavefront sensor because this allows for an assessment of the pupil condition (cataract, artificial lens, etc.) and provides a good indicator of the expected AO imaging quality in individual subjects. Moreover, pupil tracking can be implemented using these images, which further minimizes influences of eye motion on the measurements.

In our representative AO-OCT data, microstructural details of the neurosensory retina such as vessel walls and microglia cells are visualized in single volume data and over an extended field of view in healthy as well as pathological retinas. Changes in these retinal features, for example in the thickness of vessel walls or the presence of microglia, may serve as early biomarker for diseases such as diabetic retinopathy.

## 5. Conclusion

In conclusion, we introduced a novel method for focus shifting in AO-OCT imaging. The imaging results are very promising and the technique is transferable to any other AO system that is based on wavefront sensing. We further demonstrated an extension of the high resolution field of view through exploitation of faster adaptive optics along the slow scanning direction. This concept can be translated to other AO systems including SLO provided that the wavefront sensing beam is scanned similarly as the imaging beam and the AO correction speed is sufficiently high.

## Supplemental information

Supplement 1 Supplemental document https://doi.org/10.6084/m9.figshare.26788006

Visualization 1 En-face fly-through focus posterior layers, healthy volunteer https://doi.org/10.6084/m9.figshare.26784715

Visualization 2 En-face fly-through focus anterior layers, healthy volunteer https://doi.org/10.6084/m9.figshare.26784724

Visualization 3 Fly-through B-scan movie focus anterior layers, healthy volunteer https://doi.org/10.6084/m9.figshare.26784733

Visualization 4 En-face fly-through focus anterior layers, patient with diabetic retinopathy https://doi.org/10.6084/m9.figshare.26784739

## Data Availability

Data underlying the results presented in this paper are not publicly available at this time but may be obtained from the authors upon reasonable request.

## Supplemental document

See Supplement 1 for supporting content.
