# Supplementary material for: Improvements on speed, stability and field of view in adaptive optics OCT for anterior retinal imaging using a pyramid wavefront sensor
Source: Biomed Opt Express. 2024 Sep 30;15(10):6098–116. doi: 10.1364/BOE.533451 (PMC11482182; doi:10.1364/BOE.533451)
Supplement: Supplementary file 1 [file boe-15-10-6098-s001.pdf]

Supplemental Document

Biomedical Optics EXPRESS

Improvements on speed, stability and field of view in adaptive optics OCT for anterior retinal imaging using a pyramid wavefront sensor: supplement

ELISABETH BRUNNER,[1,*] LAURA KUNZE,[2] VICTORIA LAIDLAW,[3] DANIEL JODLBAUER,[4] WOLFGANG DREXLER,[1] RONNY RAMLAU,[3] ANDREAS POLLREISZ,[2] AND MICHAEL PIRCHER[1]

[1] *Center for Medical Physics and Biomedical Engineering, Medical University of Vienna, Waehringer Guertel 18-20, A-1090 Wien, Austria*
[2] *Department of Ophthalmology and Optometry, Medical University of Vienna, Waehringer Guertel 18-20, A-1090 Wien, Austria*
[3] *Johannes Kepler University Linz, Industrial Mathematics Institute, Altenbergerstraße 69, A-4040 Linz, Austria*
[4] *Johann Radon Institute for Computational and Applied Mathematics, Altenbergerstraße 69, A-4040 Linz, Austria*
[*] *a.elisabeth.brunner@meduniwien.ac.at*

This supplement published with Optica Publishing Group on 30 September 2024 by The Authors under the terms of the Creative Commons Attribution 4.0 License in the format provided by the authors and unedited. Further distribution of this work must maintain attribution to the author(s) and the published article's title, journal citation, and DOI.

Supplement DOI: https://doi.org/10.6084/m9.figshare.26788006

Parent Article DOI: https://doi.org/10.1364/BOE.533451

Improvements on speed, stability and field of view in adaptive optics OCT for anterior retinal imaging using a pyramid wavefront sensor: supplemental document

1. DM linearity for open-loop defocus application

For the *in vivo* calibration of the target slopes for closed-loop focus shifting, the desired defocus wavefront has to be introduced by the deformable mirror (DM). The required open-loop control matrix applied in this work was computed from an influence matrix that was obtained prior to integration of the DM into the system using a custom made Shack-Hartmann WFS [1] and a defined point light source. The pseudo inverse of the influence matrix was regularized approximating the H2 norm using an embedding operator. Thus, we optimize towards a smaller second derivative of the DM surface to obtain a smooth mirror shape [2, 3].

For the assessment of the linearity of the DM, positive and negative defocus wavefronts of RMS values between 0 and 6 µm were introduced with the DM and measured in the eye pupil plane using an Optocraft SH-WFS. Per defocus level, four measurements were recorded and averaged. Between the measurements, the DM was reset before the defocus was reapplied. The resulting data plotted in Fig. S1 shows good linearity over the entire defocus range that required ~80% of the DM actuator stroke. The defocus coefficients measured in the pupil plane cover a range of approximately -3.5 to 3.5 diopters. Our results confirm earlier work that outlined good linearity of ALPAO deformable mirrors in feed-forward operation [4].

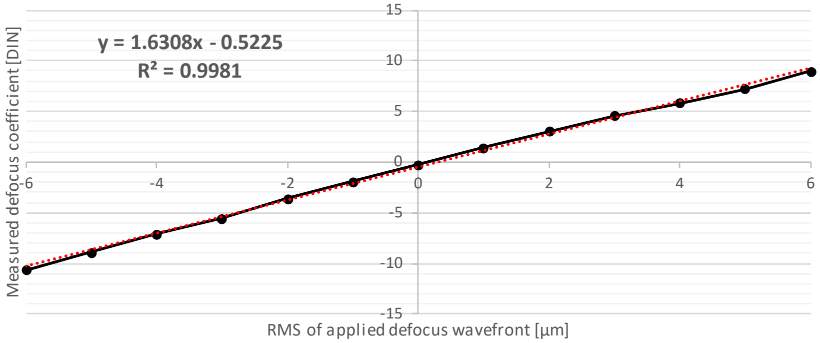

Fig. S1. The defocus coefficients (DIN / ISO 10110-5) measured in the eye pupil plane is plotted over the RMS value of the defocus wavefront applied with the deformable mirror.

2. Variation of wavefront aberration across the field of view in a model eye

The temporal evolution of RMS values of P-WFS slopes maps (cf. Section 2.3 of the main manuscript) recorded while 3D scanning provide means to investigate variations in the wavefront aberrations across the field of view. It is important to note that random fluctuations in wavefront measurements (due to sensor noise etc.) will result in random fluctuations of the RMS values, but the amplitude of these fluctuations will depend on the absolute RMS value. The presence of large absolute RMS values results in larger random RMS fluctuations (RMS noise). To remove this bias, we performed all measurements with a similar absolute RMS value. Closed-loop operation already leads to low RMS values. Thus, for the open-loop measurements we first corrected for aberrations (closing the AO loop until convergence) before opening the loop and starting the RMS measurement with a static correction in place.

In Fig. S2a), RMS time series recorded in a model eye for static aberration correction are presented. The scanning angles were set to 1° and 4°. Two different scanning scenarios, 2D scanning (slow scanner stays at the same position) and 3D scanning are investigated. The temporal evolution of aberrations under closed-loop correction (after convergence) is plotted in S2b, respectively. The AO loop rate was 25 Hz for both correction scenarios.

The time series recorded for 2D scans show only small random variations over time that are introduced by noise in the wavefront measurement. Potential fast changes in aberrations across the field of view cannot be detected, because within one WFS exposure, the beam scans several times over the field of view (>300 Hz B-scan rate, P-WFS exposure time of 20 ms) and only an averaged wavefront over the entire field of view is measured.

For 3D scans however, repeatable variations in the RMS values that are in line with the slow scanning frequency can be seen (cf. Fig. S2a). The amplitude of these variations is greatly reduced for small scanning angles. When performing closed-loop correction (cf. S2b) only residual peaks remain for the large field of view at the beginning of each 3D scan where the slow-axis scanner jumps back to its starting position and thus introduces an error in the WFS measurement.

Although all measurements were performed after prior AO correction, there are residual offsets in the absolute RMS values. To better visualize the temporal variations of aberrations, the mean RMS value of each time series was subtracted (cf. "zero mean" in Figs. S2 c) and d)) before performing a power spectra analysis (see Figs. S3-S5). For every considered setting, four power spectra were recorded and averaged.

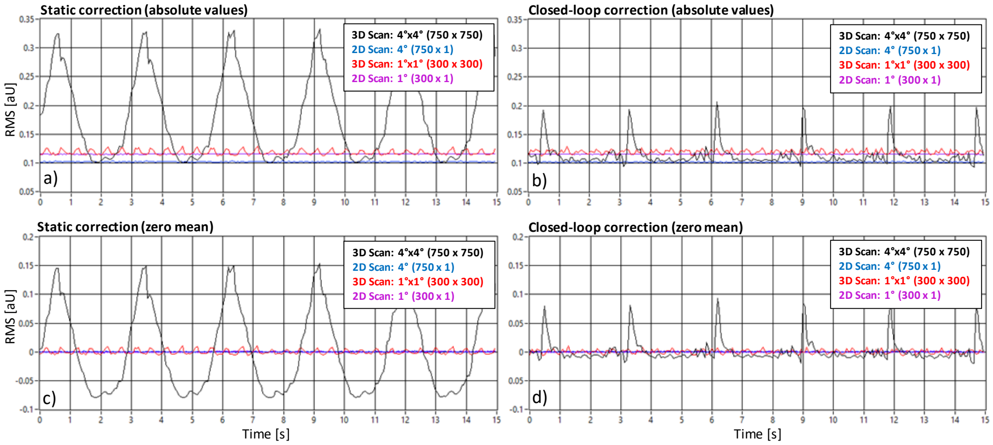

Fig. S2. Time series of RMS values of P-WFS slope maps recorded in the model eye with static correction, in **a**) and **c**) (zero mean), and with closed-loop correction, in **b**) and **d**) (zero mean). Note that the 3D scanning frequencies for the 1° and 4° field of view are 2.3 Hz and 0.4 Hz, respectively.

The difference in the power spectra between 3D and 2D scanning with a scanning angle of 4° is displayed in Fig. S3. The power spectra in Fig. S3a) show peaks corresponding to the scanning frequency of ~ 0.4 Hz of the slow scanner for both static and closed-loop correction. However, with closed-loop correction the amplitude of the peaks is reduced which is also reflected in the corresponding power rejection curve (quotient of the red and black curves in Fig. S3a) that is displayed in Fig. 3Sb). As expected, fast temporal variations in the aberrations across the field of view (introduced by the fast scanning) are neither detected nor corrected when using a 2D scan pattern (cf. Figs. S3c) and S3d)).

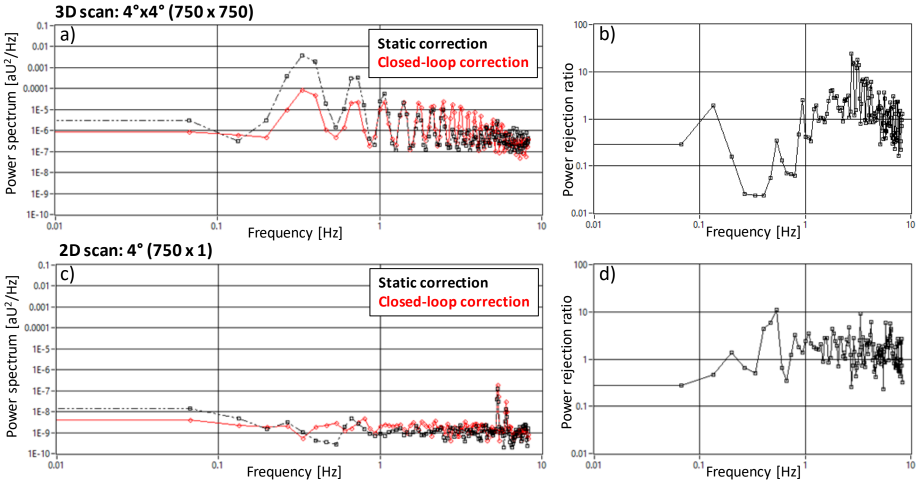

Fig. S3. Power spectra (**a, c**) and corresponding power rejection curves (**b, d**) computed for time series (18 seconds) of RMS values of P-WFS slope maps recorded in the model eye

In Fig. S4, the influence of the size of the FoV on the presence of field aberrations (i.e. aberrations that vary across the FoV) is investigated. For a better comparison, the same number of 750×750 scanning points was used for scanning angles of 1°, 2°, and 4°. The power spectra reveal temporal variations in the wavefront aberrations when the beam is moving across the FoV for all considered scanning angles. However, the amplitude of the peaks increases by at least an order of magnitude when the scanning angle is doubled.

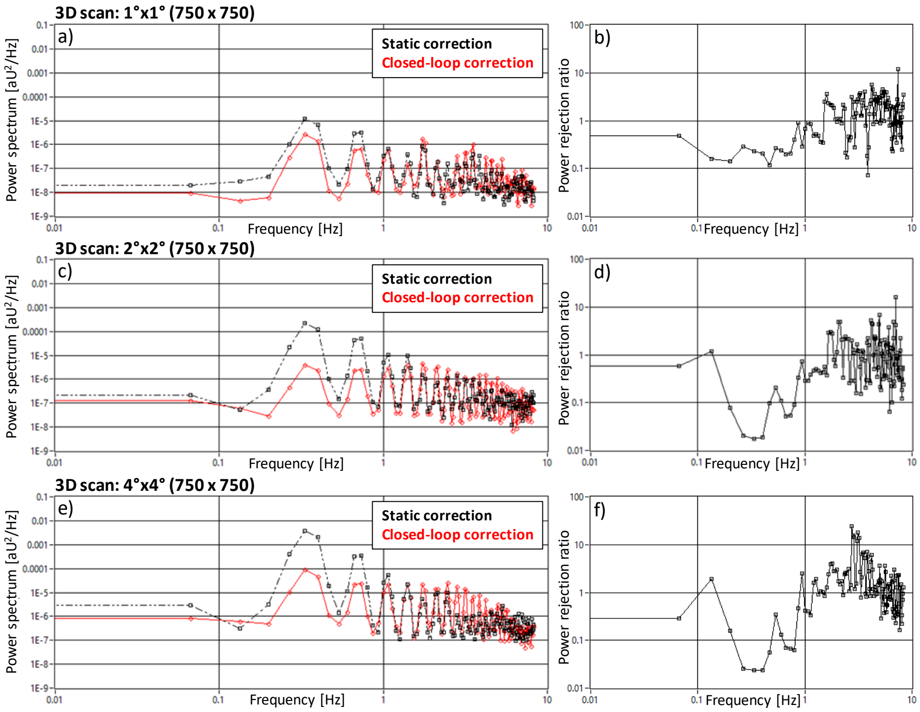

Fig. S4. Power spectra (**a, c, e**) and corresponding power rejection curves (**b, d, f**) computed for time series (18 seconds) of RMS values of P-WFS slope maps recorded in the model eye for different scan fields.

In Fig. S5, we investigate the influence of the ratio between the AO update rate and the scanning frequency along the slow axis on the ability of the AO loop to detect and compensate for temporal variations in the aberration across the FoV. Two scanning angles of 1° and 4° are considered. For an AO loop update rate of 25 Hz and a slow axis scanning frequency of 0.4 Hz, field aberrations can be detected and reduced in both the small and large FoV (cf. Figs. S5 a), b) and e), f)). For the small FoV, we further tested the 25 Hz AO loop update rate in combination with a faster slow axis scanning frequency of ~2.3 Hz. (cf. Figs. S5 c) an d)). The faster scanning prevents sufficient suppression of the field aberrations and introduces residual correction errors in the lower frequency range. For the large FoV, a slower AO loop update rate of 5 Hz was applied in combination with the slow axis scanning frequency of 0.4 Hz. Again, correction of field aberrations is less accurate with this setting and additional variations in the low frequency range are introduced by the closed loop correction (cf. Figs. S5 g), h)).

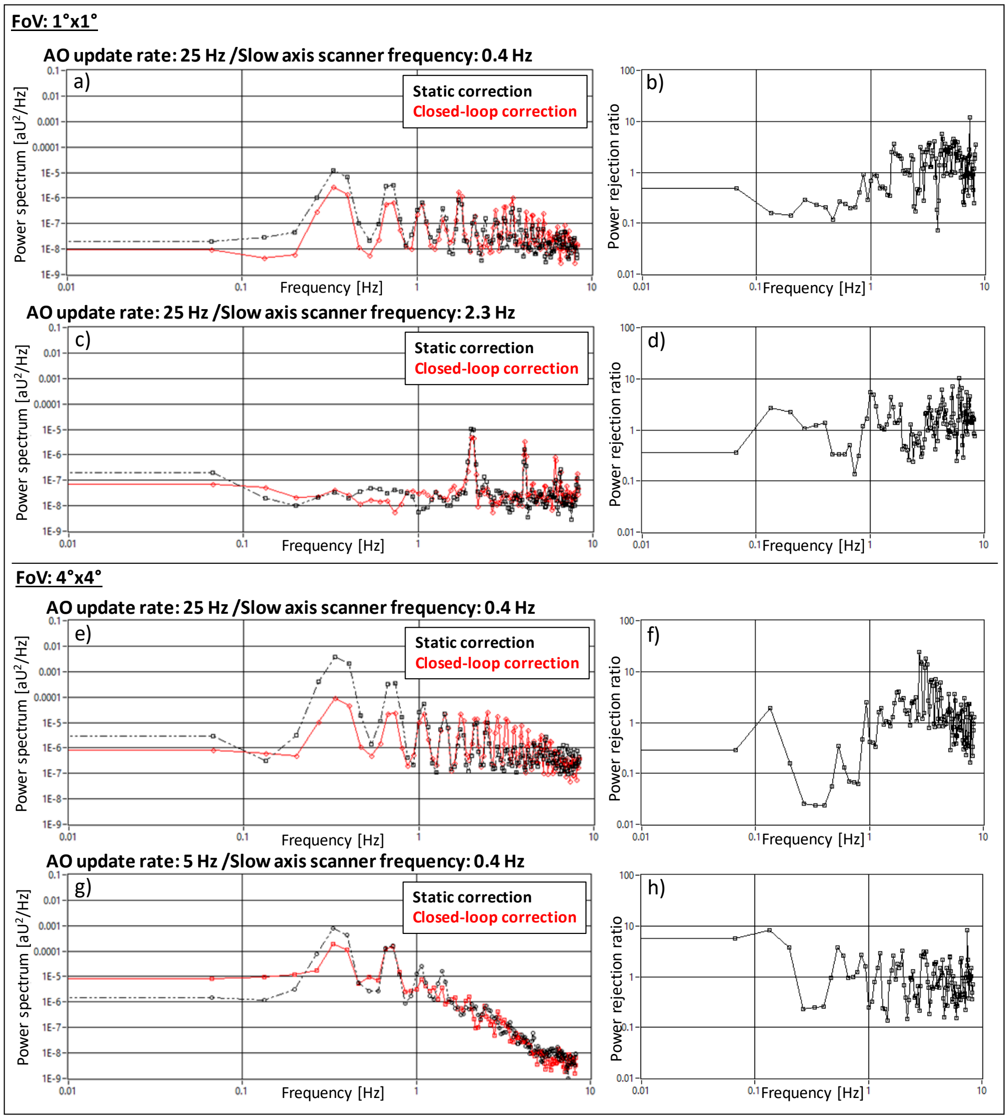

Fig. S5. Power spectra (**a, c, e, g**) and corresponding power rejection curves (**b, d, f, h**) computed for time series (18 seconds) of RMS values of P-WFS slope maps recorded in the model eye at different AO update rates and slow scanning frequencies.

3. In vivo AO-OCT performance for posterior layer imaging

The *in vivo* performance of the improved P-WFS based AO without focus shifting was assessed through AO-OCT imaging of the photoreceptor and RPE layers. Good to excellent imaging quality was achieved in all subjects. Representative data recorded at the superior vessel arc of a young healthy volunteer is presented in Fig. S6. The imaging data is extracted from the same single volume as the data presented in Fig. 5 of the main manuscript.

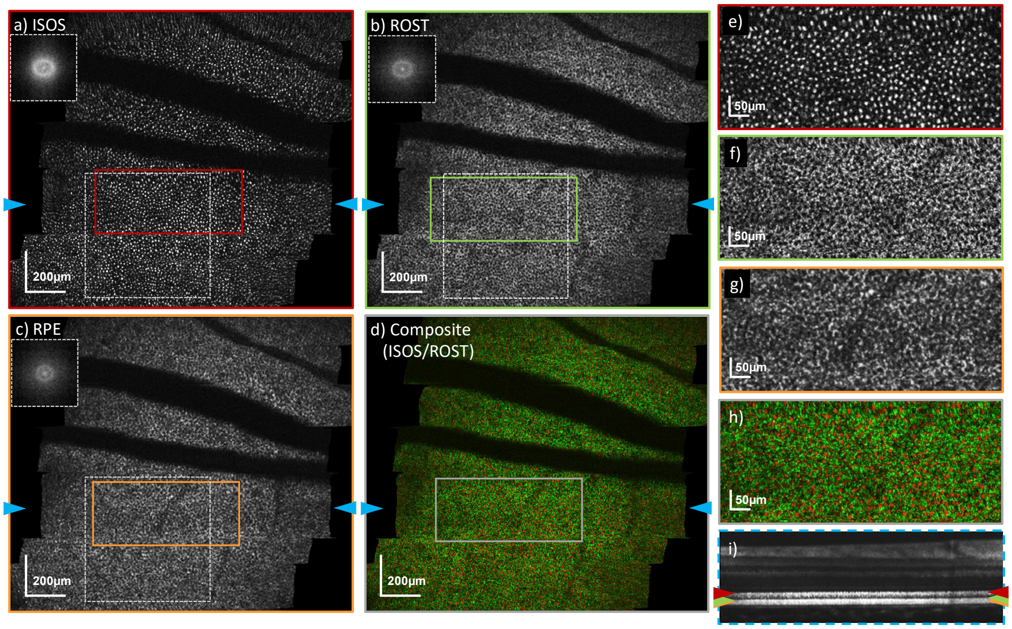

Fig. S6. Representative AO-OCT data recorded with a field of view of 4°×4° (corresponding to 1.46×1.46 mm on the retina) in the superior vessel arc of a healthy volunteer (female, 27 years, right eye, 6.38 mm pupil diameter). The en-face images in **a**)-**c**) were retrieved from a single data volume by depth integration over the junction of the inner and outer cone segments (ISOS), the rod outer segment tips (ROST) and the retinal pigment epithelium (RPE), respectively. The inserted 2D power spectra were computed for the areas indicated by gray dashed boxes. A color composite of ISOS and ROST is shown in **d**). The location of the zoom-ins shown in **e**)-**h**) are indicated with color coded boxes in **a**)-**d**). In **i**), the depth location of the enface images are indicated in an averaged B-scan.

4. Stability of closed-loop focus shifting with the P-WFS

In our standard imaging routine for extended FoV imaging (i.e. 4°×4°), two volumes are recorded in each image acquisition. We limit ourselves to two volumes, because by experience the subjects are able to stably and comfortably fixate for the resulting recording time of ~5 seconds. They tire less and it is possible to cover a larger number of focus settings and imaging locations. No significant difference was observed in AO correction quality between the first and second volumes when focus shifting was applied.

To investigate the stability of the focus shifting over a longer time span, the following three metrics were recorded for each loop iteration over a duration of 20 seconds in several patients with diabetic retinopathy (DR): the RMS value of the P-WFS slope data, the maximum positive actuator stroke applied and the maximum negative actuator stroke applied. Representative data recorded in a patient with severe DR and cataract is shown in Fig. S7. The recording was started after convergence of the AO closed-loop correction with a zero target. The following events are marked in the curves: The *in vivo* calibration of the defocus target is performed and closed-loop focus shifting is turned on. The focus shifting converges and subsequently five AO-OCT volumes with a 4°×4° FoV are recorded. All three curves show that the AO loop stays stable

over the course of ~16 seconds. In Fig. S7b), it can be observed in the maximum positive stroke how the AO correction is compensating for the field aberrations along the slow scanning axis. The slight shift in RMS value at the start of the volume recording is caused by a change of the scanning pattern. During imaging the full sampling of 750 B-scan is applied while during convergence of the closed-loop AO correction (with or without focus shifting) the sampling in the slow scanning axis is reduced.

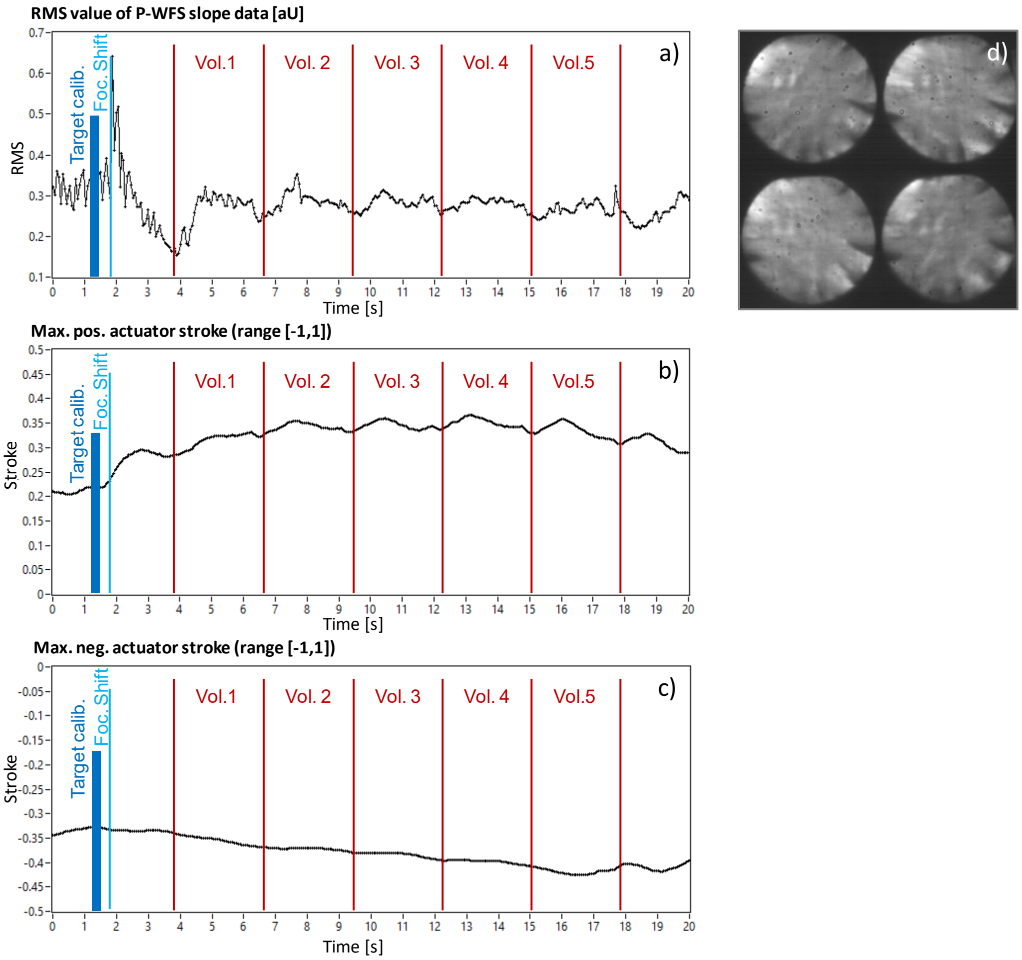

Fig. S7. Representative AO data recorded in the superior vessel arc of a patient with severe DR (68 years, male, right eye, 6.87 pupil diameter) using a FoV of 4°×4°. The curves in **a**), **b**), and **c**), respectively, show the RMS value of the P-WFS slope data, the maximum positive actuator stroke applied and the maximum negative actuator stroke applied over a duration of 20 seconds. The duration of the *in vivo* calibration of the defocus target is marked in dark blue, the moment the closed-loop shifting is turned in bright blue, and the start and end points of the 5 volume recordings in red. **d**) Pupil plane images recorded by the P-WFS during the target calibration.

References

1. M. Laslandes, M. Salas, C. K. Hitzenberger, and M. Pircher, "Increasing the field of view of adaptive optics scanning laser ophthalmoscopy," Biomed. Opt. Express **8**, 4811-4826 (2017).
2. S. Hubmer, E. Sherina, and R. Ramlau, "Characterizations of adjoint Sobolev embedding operators with applications in inverse problemsM2 116," ETNA - Electronic Transactions on Numerical Analysis, 116-144.
3. B. L. Ellerbroek, "Efficient computation of minimum-variance wave-front reconstructors with sparse matrix techniques," Journal of the Optical Society of America A **19**, 1803-1816 (2002).

4. U. Bitenc, N. A. Bharmal, T. J. Morris, and R. M. Myers, "Assessing the stability of an ALPAO deformable mirror for feed-forward operation," Opt Express **22**, 12438-12451 (2014).
